# Supplementary material for: Building and Testing PPARγ Therapeutic ELB00824 with an Improved Therapeutic Window for Neuropathic Pain
Source: Molecules. 2020 Mar 3;25(5):1120. doi: 10.3390/molecules25051120 (PMC7179195; doi:10.3390/molecules25051120)
Supplement: Supplementary file 1 [file molecules-25-01120-s001.pdf]

molecules

Supplementary Materials: Building and Testing PPARγ Therapeutic ELB00824 with an Improved Therapeutic Window for Neuropathic Pain

Karin N. Westlund and Morgan Zhang

The most beneficial characteristic of ELB00824 is not its efficacy, but the therapeutic window which is superior to other PPARγ agonists, according to available information. This is supported by the unprecedented BBB permeability of ELB00824.

Most of these models are based on computationally derived physicochemical descriptors, namely the lipophilicity (logP or logD), the topological polar surface area (TPSA). LogD is a better permeability predictor than logP, because it accounts for the pH dependence of a molecule in aqueous solution, normally at pH 7.4 (i.e., $logD_{7.4}$). In almost all these models, high logBB values are favored by low MW values, high $logD_{7.4}$ values, low TPSA values, [1]. TPSA defined as the surface area occupied by nitrogen and oxygen atoms and the polar hydrogens attached to them, and the less the sum of the nitrogen and oxygen (N+O), the lower TPSA. So the high logBB values are also favored by low (N+O). In addition, high logBB values are favored by non-ionizable molecules, where logD7.4 = logP. For the singly ionized acid species, where $logD_{7.4}$ = logP – log[1+10^(7.4-pKa)] [2], the lower is the pKa value, the lower is $logD_{7.4}$ value. Therefore, high logBB values are <u>not</u> favored by lower pKa value of acid groups. Examples of these models are as follows [3]: logPS = -2.19 + 0:262 * $logD_{7.4}$ + 0.0683*vsa_base – 0.009*TPSA, where vsa_base is the van der Waals' surface area due to basic atoms.

Table 1 shows that the logBB and logPS value of ELB00824 is highest (0.747 and -0.79, respectively), indicating that ELB00824 is the compound with excellent BBB permeability. A series of unique properties of ELB00824, including low MW, high logD7.4, non-ionizable, extremely low TPSA and (N+O), lead its excellent BBB permeability.

Reference:

1. Clark D.E. Chapter 10 Computational Prediction of ADMET Properties: Recent Developments and Future Challenges, Annual Reports in Computational Chemistry. 2005, 1: 133-51.
2. Xing L., and Glen R.C. Novel methods for the prediction of logP, pKa, and logD. J. Chem. Inf. Comput. Sci. 2002, 42: 796–805
3. Liu X., Tu M., Kelly R.S., Chen C., Smith B.J. Development of a computational approach to predict blood–brain barrier permeability, Drug Metab. Dispos., 2004, 32, 132–139.
4. https://en.wikipedia.org/wiki/Sodelglitazar. Accessed February 2020.

Table S1. Structures of the PPARγ agonists listed in Table 1.

Name	Structure	Log BB	Name	Structure	Log BB
**ELB00824**	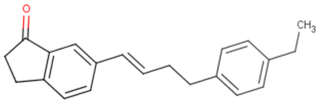	0.747	**Sodelglitazar**	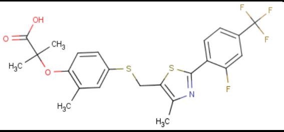	0.408
**VCE-004.8**	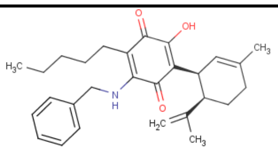	0.208	**Arhalofenate**	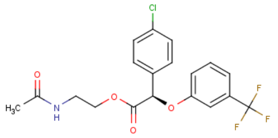	0.178
**Astaxanthin**	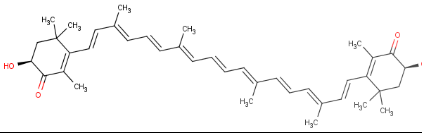	0.036	**Netoglitazone**	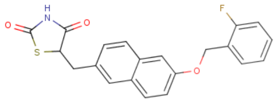	0.030
**Saroglitazar**	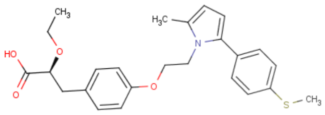	-0.030	**GED-0507-34-levo**	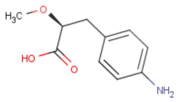	-0.074
**Tetradecylthioacetic acid**	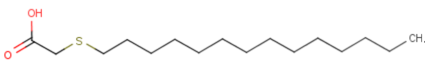	-0.148	**Norbixin. Macuneos**	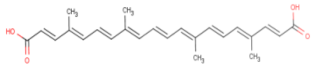	-0.177
**Daidzein**	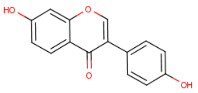	-0.182	**Chiglitazar**	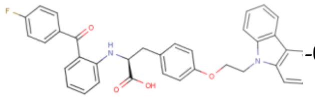	-0.223
**Oxeglitazar**	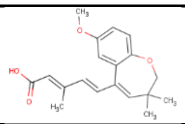	-0.287	**Farglitazar**	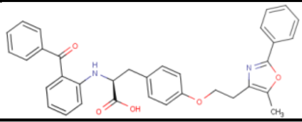	-0.289
**GSK-376501**	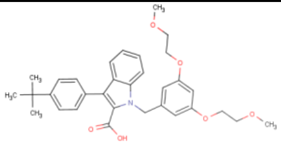	-0.343	**DSP-8658**	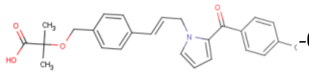	-0.429
**MK-0767**	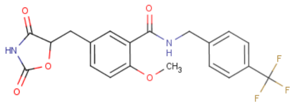	-0.430	**Etalocib**	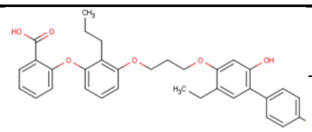	-0.516
**FK-614 ,ATx08-001**	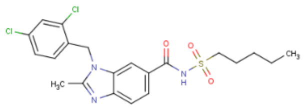	-0.542	**OMS-403, Pioglitazone**	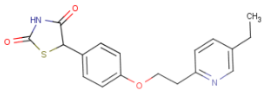	-0.561
**10-Nitrooctadec-9-enoic acid**	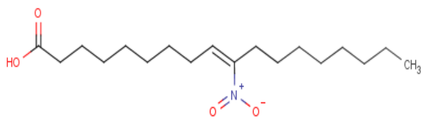	-0.564	**Troglitazone**	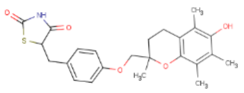	-0.567
**Rivoglitazone**	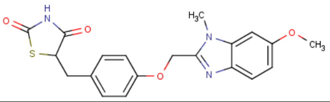	-0.573	**Peliglitazar**	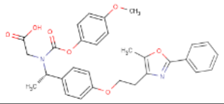	-0.582
**Imiglitazar**	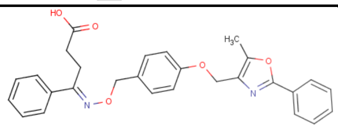	-0.604	**Efatutazone**	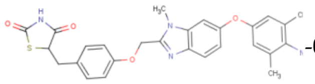	-0.672

**Mesalazine**	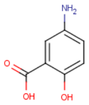	-0.689	**MN-102**	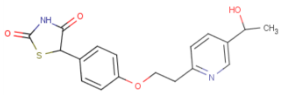	-0.714
**T3D-959**	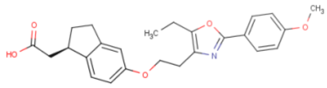	-0.718	**Ragaglitazar**	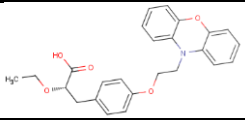	-0.718
**Darglitazone**	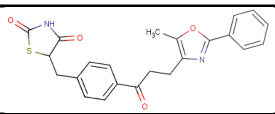	-0.722	**Rosiglitazone**	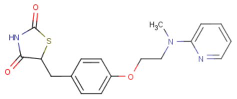	-0.727
**Reglitazar**	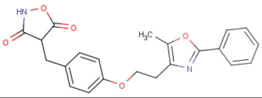	-0.751	**Muraglitazar**	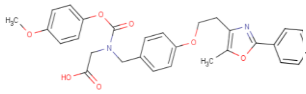	-0.778
**Naveglitazar**	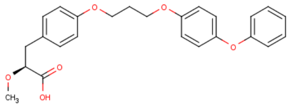	-0.833	**Edaglitazone**	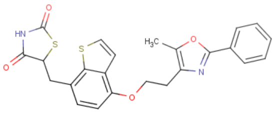	-0.872
**LY-510929**	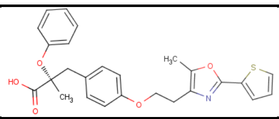	-0.877	**Balaglitazone**	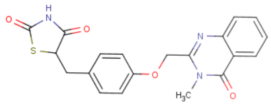	-0.906
**Aleglitazar**	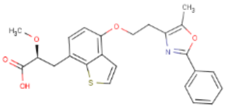	-0.958	**Indeglitazar**	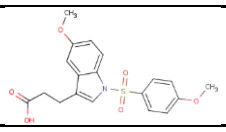	-1.018
**E-3030**	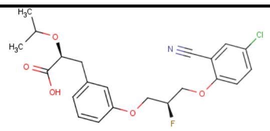	-1.089	**DS-6930**	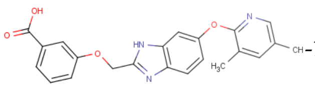	-1.128
**Tesaglitazar**	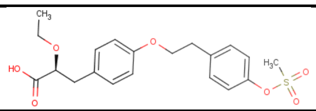	-1.136	**CHS 131**	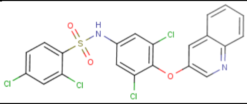	-1.167
**Sipoglitazar**	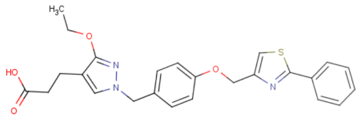	-1.230	**Lanifbranor**	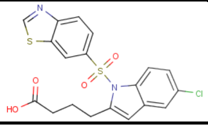	-1.327
**CLX-0921**	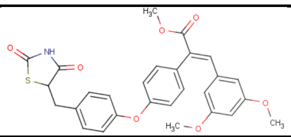	-1.331	**MK-0533**	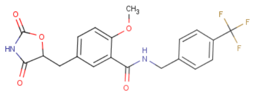	-1.362
**Lobeglitazone**	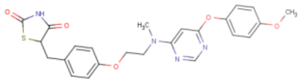	-1.466	**Cevoglitazar**	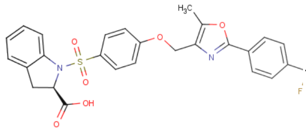	-1.592

The structures and PPARγ activity information were found in the following references:

1. Hong F, Xu P, Zhai Y. et al. The Opportunities and Challenges of Peroxisome Proliferator-Activated Receptors Ligands in Clinical Drug Discovery and Development. Int J Mol Sci. 2018;19(8). pii: E2189.
2. Cheng HS, Tan WR, Low ZS, et al. Exploration and Development of PPAR Modulators in Health and Disease: An Update of Clinical Evidence. Int J Mol Sci. 2019 Oct 11;20(20). pii: E5055.
3. https://www.medchemexpress.com/Targets/PPAR.html
4. A R, Agrawal N, Kumar H, et al. Norbixin, an apocarotenoid derivative activates PPARγ in cardiometabolic syndrome: Validation by in silico and in vivo experimental assessment. Life Sci. 2018; 209: 69-77.
